# Changing practice patterns in axillary management for patients with node-positive breast cancer towards increased use of sentinel lymph node biopsy-alone after neoadjuvant chemotherapy: results of a survey (MF17-01) among Turkish surgeons

**DOI:** 10.1007/s00423-025-03767-9

**Published:** 2025-06-16

**Authors:** Neslihan Cabıoğlu, Damla Okan Ercan, İrem Karataş, Erhan Eröz, Safa Toprak, Selman Emiroğlu, Elnur Hüseynov, Enver Özkurt, Baran Mollavelioğlu, Mustafa Tükenmez, Mahmut Müslümanoğlu, Abdullah İğci, Vahit Özmen

**Affiliations:** 1 Istanbul Faculty of Medicine, Department of General Surgery, Istanbul University, Istanbul, Türkiye; 2 Present Address: Department of General Surgery, Mardin Training and Research Hospital, Mardin, Türkiye; 3 Present Address: Department of General Surgery, Sakarya University Training and Research Hospital, Sakarya, Türkiye; 4 Present Address: Department of General Surgery, Koç University School of Medicine, Istanbul, Türkiye; 5 Present Address: Department of General Surgery, Avrupa Şafak Hospital, Istanbul, Türkiye; 6 Present Address: Department of General Surgery, School of Medicine, Demiroğlu Bilim University, Istanbul, Türkiye; 7 Present Address: Breast Center, American Hospital, Istanbul, Türkiye; 8 Present Address: Breast Center, Istanbul Florence Nightingale Hospital, Istanbul, Türkiye; 9 Istanbul Faculty of Medicine, Department of General Surgery, Istanbul University, Çapa Millet cad, Fatih, 34093 Istanbul, Türkiye

**Keywords:** Sentinel lymph node biopsy, Axillary dissection, Neoadjuvant chemotherapy, Axillary recurrence, Locoregional recurrence, Regional nodal irradiation, Breast cancer recurrence, Survival, Young age, Non-luminal pathology

## Abstract

**Background:**

This study aimed to determine the knowledge of major benchmark trials among Turkish general surgeons to investigate if they have adopted the results in their practice.

**Methods:**

A total of 101 general surgeons from the Turkish Federation of Breast Diseases Society (TFBDS) were asked to complete a survey that included 24 multiple-choice questions regarding the surgical practice in axillary surgery for early and locally advanced breast cancer.

**Results:**

Most surgeons were familiar with prospective axillary surgery studies including ACOSOG Z0011 (*n* = 77, 76.2%), AMAROS (*n* = 76, 75.2%), IBCSG 23 − 01 (*n* = 58, 57.4%), ACOSOG Z1071 (*n* = 63, 62.4%), and SENTINA (*n* = 67, 66.3%). Among the surgeons participating in the present survey, breast surgeons (38.6%) were less likely to perform axillary lymph node dissection (ALND) in early stage patients with a 1–2 positive sentinel lymph node biopsy (SLNB) with micro- or macrometastases, as opposed to those who defined themselves as general surgeons (ALND; 36.8% vs. 63.9%, *p* = 0.015). Almost all surgeons suggested neoadjuvant chemotherapy (NAC) for patients presenting with T4 (94.8%) or N2-3 disease (92.0%), whereas almost half of the surgeons (40.5%) always proceeded with NAC in patients with clinically node-positive cN1 breast cancer. Overall, 86.1% of surgeons performed SLNB in patients whose axilla became clinically negative after NAC. More than half of the surgeons (55.2%) preferred blue dye as the SLNB technique and 37 (42.5%) used the combined method. Among 87 surgeons, 24.1% (*n* = 21) always, 39.1% (*n* = 34) sometimes, and 36.8% (*n* = 32) never preferred clip marking of axillary metastatic lymph nodes before NAC, whereas 56.4% performed targeted axillary dissection (TAD) after NAC. In cN+ patients before NAC, the majority of surgeons (74.3%) did not perform ALND in patients with at least three lymph nodes removed and SLNB negative. Of note, more than half of the surgeons (51.5%) did not perform ALND in the presence of isolated tumor cells or micrometastases among the three SLNs as long as regional nodal irradiation was received. However, 54.5% of the patients routinely underwent ALND in the presence of macrometastatic residual nodal disease after NAC.

**Conclusion:**

Deescalating strategies in axillary surgery have been increasing in both initially clinically node-negative and-positive breast cancers as long as nodal radiation is provided.

## Introduction

Axillary surgical interventions are important for staging and predicting breast cancer prognosis. This is a proven guide for making local, regional, and systemic treatment decisions. As we have a better understanding of the nature of breast cancer in the last decade, it has been shown that axillary dissection does not have a significant impact in preventing distant metastasis, as previously thought. According to the results of the NSABP B-04 study, there was no statistically significant increase in distant organ metastasis or breast cancer-related mortality in patients with lymph node positivity who didn’t receive adjuvant radiotherapy or didn’t undergo an axillary dissection [1]. In the last decade, sentinel lymph node biopsy (SLNB) has replaced axillary lymph node dissection (ALND) in early breast cancer with clinically and pathologically node-negative disease [2].

The ACOSOG Z0011 trial revealed that ALND has no impact on locoregional recurrence or overall survival in selected patients with T1-T2 tumors with 1–2 SLNB positivity who underwent breast conservation therapy with whole-breast irradiation [3]. The International Breast Cancer Study Group (IBSCG) 23 − 01 study published after the ACOSOG Z0011 trial in 2013 showed that axillary dissection was not necessary for patients with micrometastases [4]. Furthermore, results of the AMAROS trial published in 2014 revealed in SLNB(+) cases that radiotherapy was equally effective as axillary dissection for local control of the axilla [5]. Both AMAROS and IBSCG 23 − 01 studies included patients who underwent mastectomy, which distinguished them from the ACOSOG Z0011. The long-term findings of these trials also supported the deescalation of axillary surgery in favor of SLNB due to the decreased morbidity of ALND compared to SLNB, despite similar oncological outcomes in clinically node-negative breast cancer [6–9].

According to the results of ACOSOG Z1071 [10] and SENTINA [11], axillary interventions have also changed in patients with clinically node-positive (cN+) breast cancer after neoadjuvant chemotherapy (NAC). Consequently, more conservative approaches such as omitting ALND were indicated to increase the quality of life by reducing overall surgical morbidity in selected patients with a good response to chemotherapy without compromising oncological safety [12–15]. Accepting new modalities and dropping old habits can be extremely slow among surgeons, even in well-established, highly respected centers. This study aimed to determine the knowledge of the major benchmark trials among Turkish general surgeons and to investigate whether they have adopted the results in their surgical practice.

## Methods

A survey was developed to evaluate surgeon awareness of the ACOSOG Z0011, AMAROS, IBCSG 23 − 01, ACOSOG Z1071, and SENTINA trial results and to determine how these results have impacted the surgical management of breast cancer in Türkiye. Institutional ethical approval was obtained from the Istanbul University Istanbul Faculty of Medicine Ethical Commettee. The survey consisted of 24 multiple-choice questions in two parts: early stage and locally advanced breast cancer. This survey study regarding surgical treatment of the axilla was conducted among 101 general surgeons who were members of the Turkish Federation of Breast Diseases Society (TFBDS) and responsible for breast cancer surgery at their institutions and/or hospitals. Participants were divided into groups according to the type of institution they worked for, their daily surgical practice in general surgery, breast surgery and surgical oncology cases, number of patients treated in the institution per year, and rate of patients receiving neoadjuvant chemotherapy. Questions included assessments of the respondents’ surgical practice, familiarity with prospective trials in axillary surgery, and preferences in the surgical management of patients with positive SLNs with low-volume metastases in early breast cancer, and initially clinically node-positive axilla after NAC.

The SPSS 25 software program (Statistical Package for Social Sciences; SPSS, IBM Corp., Armonk, NY, USA) was used in statistical analyses to perform the descriptive analyses. Categorical variables, including associations between surgeons’ institutions or their specialization in breast surgery and knowledge about important benchmark studies in axillary surgery, were evaluated using the chi-square test. A p-value ≤ 0.05 was considered statistically significant.

## Results

One hundered one surgeons participated in this survey. The surgeons’ characteristics are listed in Table 1. When participants were asked to describe their daily practice based on the cases they performed, all participants (100%, *n* = 101) reported performing general surgery cases beyond surgical oncology and breast surgery. Additionally, 38.6% (*n* = 39) reported performing breast surgery cases, while 12.8% (*n* = 13) reported performing surgical oncology cases. (Table 1). The majority (50.5%) of surgeons were affiliated with university hospitals and worked as academic faculties. Overall, 63 (63%) of the general surgeons worked in an institution with > 100 breast surgeries per year. Furthermore, 32 (31.7%) performed individualy > 100 breast cancer surgeries per year, whereas 69 (68.3%) had ≤ 100 cases with breast cancer surgery. Overall, 68.4% of surgeons had ≥ 10 years of experience in breast cancer surgery. Most surgeons were familiar with prospective axillary surgery studies including ACOSOG Z0011 (*n* = 77, 76.2%), AMAROS (*n* = 76, 75.2%), IBCSG 23 − 01 (*n* = 58, 57.4%), ACOSOG Z1071 (*n* = 63, 62.4%), and SENTINA (*n* = 67, 66.3%) (Table 2). Surgeons affiliated with public, and private universities and training hospitals were more likely to be familiar with the above-mentioned axillary surgery trials than those working at public hospitals (Table 2). Similarly, surgeons who defined themselves as breast surgeons were more likely to know all of these prospective studies than surgeons who did not define themselves as breast surgeons (Table 2).

**Table 1:** Characteristics of surgeons who participated in the survey and their affiliated institutions or hospitals (*n* = 101)

		*n*	%
Practice type	*General Surgery*	101	100
	*Breast Surgery*	39	38.6
	*Surgical Oncology*	13	12.8
Affillation	*Public University*	41	40.6
	*Private University*	10	9.9
	*Training and Research Hospital*	21	20.8
	*Public Hospital*	13	12.9
	*Private Hospital*	16	15.8
Number of surgeons performing breast surgery at each institution (*n* = 100)	*1*	16	16.0
	*2*	25	25.0
	*≥ 3*	59	59.0
Experience with breast surgical oncology (year)	*0–5*	18	17.8
	*5–9*	14	13.9
	*10–15*	15	14.9
	*> 15*	54	53.5
Number of breast cancer patients treated at the institution per year (*n* = 100)	*0–50*	19	19.0
	*50–100*	18	18.0
	*100–200*	23	23.0
	*200–300*	18	18.0
	*> 300*	22	22.0
Number of breast cancer patients treated by each surgeon per year	*0–50*	37	36.6
	*50–100*	32	31.7
	*100–200*	18	17.8
	*200–300*	9	8.9
	*> 300*	5	5.0
Number of breast cancer patients treated with surgery after neoadjuvant chemotherapy at each institution per year	*0–15*	30	29.7
	*15–30*	20	19.8
	*30–50*	11	10.9
	*> 50*	16	15.8
	*50–100*	24	23.8
Number of breast cancer patients treated with surgery after neoadjuvant chemotherapy by the surgeon at each institution per year (*n* = 100)	*0–15*	45	45.0
	*15–30*	24	24.0
	*30–50*	9	9.0
	*> 50*	10	10.0
	*50–100*	12	12.0

**Table 2:** The familiarity of the surgeons with the prospective scientific studies on the axilla

		Sufficient level of knowledge				
		ACOSOG Z0011	AMAROS	IBCSG 23 − 01	ACOSOG Z1071	SENTINA
Institution	n	n(%)	n(%)	n(%)	n(%)	n(%)
Total		77 (76.2)	76 (75.2)	58 (57.4)	63 (62.4)	67 (66.3)
Public University	41	35(85.4)	36(87.8)	31(75.6)	30(73.2)	31(75.6)
Private University	10	9(90.0)	10(100.0)	7(70.0)	8(80.0)	9(90.0)
Public and Training Hospital	21	14(66.7)	12(57.1)	9(42.9)	12(57.1)	13(61.9)
Public Hospital	13	5(38.5)	6(46.2)	2(15.4)	4(30.8)	5(38.5)
Private Hospital	16	14(87.5)	12(75.0)	9(56.3)	9(56.3)	9(56.3)
*P-value*		*p* = 0.004	*p* = 0.003	*p* = 0.002	*p* = 0.05	*p* = 0.049
Practice type						
Breast Surgeon	39	38(97.4)	37(94.9)	32(82.1)	33(84.6)	34(87.2)
General Surgeon	62	39(62.9)	39(62.9)	26(41.9)	30(48.4)	33(53.2)
*P-value*		*p* < 0.001	*p* < 0.001	*p* < 0.001	*p* < 0.001	*p* < 0.001

*p* < 0,05; Chi-Square Test

Among the surgeons who participated in the present survey, breast surgeons were less likely to perform ALND in early stage patients with–1–2 positive SLNB with micro- or macrometastases, as opposed to those who defined themselves as general surgeons (ALND; *n* = 14, 36.8% vs. *n* = 39, 63.9%, *p* = 0.015, Table 3). However, the majority of surgeons (89.9%) still used intraoperative pathological evaluation (Table 4).

**Table 3:** Management of the axilla according to the institutional affiliation type and surgical practice type (*n* = 99)

		Axillary management		
		Performing SLNB in cN0 patients after NAC	Always do axillary marking before NAC	ALND in an early stage pNmic&pN1 patient
Institution	n	n(%)	n(%)	n(%)
Public University Hospital	40	38(95.0)	10(25.0)	17(42.5)
Private University Hospital	10	10(100.0)	5(50.0)	4(40.0)
Training and Research Hospital	21	15(71.4)	2(9.5)	15(71.4)
Public Hospital	12	10(83.3)	1(8.3)	9(75.0)
Private Hospital	16	14(87.5)	4(25.0)	8(50.0)
*P*-value		*p* = 0.065	*p* = 0.020*	*p* = 0.103
Practice type				
Breast Surgeon	38	35(92.1)	13(34.2)	14(36.8)
General Surgeon	61	52(85.2)	9(14.8)	39(63.9)
*P*-value		*p* = 0.362	*p* = 0.076	*p* = 0.015

*SLNB* Sentinel lymph node biopsy, *ALND* axillary lymph node dissection, *NAC* neoadjuvant chemotherapy

**Table 4:** Management of the axilla among Turkish surgeons in patients with early stage and locally advanced breast cancer

		*n*	%
Always recommended NAC for patients	For T4 tumors	96	94.8
	For N2-3 disease	93	92.0
	For clinically node-positive (cN1) breast cancer	41	40.5
cT1-2 N0:			
Situations where ALND will not be performed in a patient who has a total of 2–3 SLNs removed and will receive RT(either to the breast or to the axillary region) (*n* = 101)	*Micrometastasis are detected in a sentinel lymph node*	58	57.4
	*Micro or macrometastases are detected in a sentinel lymph node*	40	39.6
	*Micrometastases are detected in at most 2 sentinel lymph nodes*	44	43.6
	*Micro and/or macrometastases are detected in at most 2 sentinel lymph nodes*	47	46.5
Intraoperative pathological examination of sentinel lymph nodes (*n* = 99)	*Always*	76	76.77
	*Most of the time*	13	13.13
	*Sometimes*	5	5.05
	*Never*	5	5.05
cN-positive:			
Fine-needle aspiration biopsy (FNAB) or core needle biopsy of the suspicious lymph nodes in axillary USG in locally advanced breast cancer (LABC) (*n* = 98)	*Always*	52	53.1
	*If the lymph node to be marked with a clip*	18	18.4
	*If the physical examination is metastatic and the lymph node will not be marked with a clip*,* I wont*	14	14.3
	*Never*	14	14.3
SLNB for cN(+) patients who became clinically negative after NAC (*n* = 101)	*Yes*	87	86.1
	*No*	14	13.9
SLNB technique (*n* = 87)	*Blue dye*	48	55.2
	*Lymphoscintigraphy after radioisotope injection*	2	2.3
	*Dual method*	37	42.5
Approach to the removal of the lymph nodes (*n* = 87)	*Removal of the the sentinel lymph node alone*	5	5.7
	*Removal of the sentinel lymph node along with the additional clinically suspicious lymph nodes*	82	94.3
Number of SLNs/LNs removed (*n* = 87)	*At least 1*	2	2.3
	*At least 2*	24	27.6
	*At least 3*	61	70.1
Clipping of axillary metastatic lymph nodes before NAC (*n* = 87)	*Always*	21	24.1
	*Sometimes*	34	39.1
	*Never*	32	36.8
Veryfying the removal of the clip-marked lymph node in a patient who was operated after NAC by specimen radiography (*n* = 55)	*Yes*	32	58.2
	*No*	6	10.9
	*Sometimes*	17	30.9
Targeted axillary dissection for retrieving the clip-marked lymph node with methods such as wire or ROLL (*n* = 55)	*Yes*	31	56.4
	*No*	24	43.6
Omitting ALND after NAC in a clinical node-positive breast cancer patient (*n* = 101)	*Pathologically negative SLNB (number of SLNs does not matter)*	43	42.6
	*Pathologically negative SLNB (at least 3 LNs removed)*	75	74.3
	*When isolated tumor cells or micrometastases are detected in the SLNB paraffin block (at least 3 LNs have been removed)if receiving nodal irradiation*	52	51.5
	*Patients without additional pathologically/clinically metastatic non-sentinel lymph nodes*,* in case with pathologically positive SLNB if receiving nodal irradiation*	21	20.8
	*I perform low axillary dissection (level 1) in cases with SLNB positive but no additional clinically/pathologically metastatic lymph nodes if receiving nodal irradiation*	15	14.9
ALND after NAC in a clinical node-positive breast cancer patient* (*n* = 101)	*I always do axillary dissection*,* no matter SLNB is negative or positive*	3	3.0
	*If 1 SLN is removed*,* I do ALND even if the SLN is negative*	10	9.9
	*Every SLNB positive case (isolated tumor cell or micro or macrometastasis) despite regional nodal irradiation*	31	30.7
	*If SLNB contains macrometastasis*,* I do ALND despite regional nodal irradiation*	55	54.5
	*If SLNB contains macrometastasis*,* I perform level 1 low axillary dissection to include clinically suspected lymph nodes despite regional nodal irradiation*	38	37.6

*LN* lymph node, *SLN* sentinel lymph node, *SLNB* Sentinel lymph node biopsy, *ALND* axillary lymph node dissection, *NAC* neoadjuvant chemotherapy, *ROLL* radioactive occult lesion localisation

*:More than one option is marked

Among the 101 surveyed surgeons, 94.8% (*n* = 96) consistently recommended neoadjuvant chemotherapy (NAC) for patients with T4 tumors, while 92.0% (*n* = 93) did so for patients with N2-3 disease. In contrast, 40.5% (*n* = 41) of surgeons always proceeded with NAC for patients with clinically node-positive (cN1) breast cancer. Overall, 87 (86.1%) surgeons performed SLNB in patients whose axilla became clinically negative after NAC. Notably, more than half of the surgeons (55.2%) preferred blue dye as the SLNB technique, and 37 (42.5%) used the combined method. Furthermore, 82 (94.3%) surgeons removed clinically suspicious lymph nodes in addition to sentinel lymph nodes. Among the 87 surgeons who responded regarding the marking of axillary metastatic lymph nodes before NAC, 24.1% (*n* = 21) always preferred marking with a clip, 39.1% (*n* = 34) sometimes preferred it, and 36.8% (*n* = 32) never opted for clip marking. (Table 4). Surgeons affiliated with university hospitals were more likely to proceed with axillary marking of the cN+ positive before neoadjuvant treatment and perform SLNB in patients who became clinically node-negative after NAC compared to those surgeons from public hospitals (Table 3).

Surgeons were asked under what conditions they would refrain axillary dissection in patients with clinically node-positive breast cancer before NAC. The majority of surgeons (86.1%) indicated that they would perform SLNB in patients achieving a clinical complete response after NAC, whereas 74.3% reported that they wouldn’t perform ALND in patients with at least three lymph nodes removed and a negative SLNB result. Interestingly, surgeons with more than 10 years of experience were more likely than those with less experience to avoid ALND in cN+ patients who were ypN0 after SLNB after NAC, regardless of the number of SLNs removed or the number of positive lymph nodes before NAC (ALND; 34.8% vs. 59.4%, *p* = 0.035).

Overall, more than half of the surgeons (51.5%) preferred not to perform ALND if at least 3 sentinel lymph nodes (SLN) were removed and isolated tumor cells (ITCs) or micrometastases were detected in paraffin blocks, provided regional nodal irradiation (RNI) was ensured. Of note, 21 surgeons (20.8%) indicated that they wouldn’t perform ALND if there was no extensive lymph node involvement in the preoperative radiological findings, at least three nodes were removed, and SLNB was positive. However, 30.7% of surgeons opted for ALND in all patients with nodal metastases, regardless of metastasis type (ITC, micrometastasis, or macrometastasis). Additionally, 54.5% of surgeons preferred to perform ALND consistently in patients with macrometastasis. Furthermore, 15 surgeons (14.9%) favored level 1 low axillary dissection in patients with positive SLN and no additional clinical lymph nodes. Few surgeons (3%) surgeons proceed with ALND without performing SLNB in patients who had node-positive disease before NAC (Table 4).

## Discussion

In the present study, a survey was conducted to evaluate the surgeon’s familiarity with published prospective trials, including the ACOSOG Z0011, AMAROS, IBCSG 23 − 01, ACOSOG Z1071, and SENTINA trials, which are important in the management of the axilla in patients with early and locally advanced breast cancer surgery. We also aimed to assess whether their knowledge has led to a change in their daily surgical practice, and how these results have impacted the surgical management of breast cancer in Türkiye. A total of 101 general surgeons who were members of the Turkish Federation of Breast Diseases Society completed the questionnaire. Although all the general surgeons who participated in this survey were responsible for breast cancer surgery at their institutions or hospitals, only 38.6% of the participating general surgeons defined themselves as breast surgeons. Briefly, findings from the present study showed that the majority of surgeons were familiar with these studies. Participants of this survey reported knowledge of ACOSOG Z0011 with a percentage of 76.2% with decreasing knowledge about other studies as IBCSG 23-01was the least known (57.4%). This could also be due to the prior publication date of ACOSOG Z0011 among other studies asked in the present survey.

A similar survey was performed among 849 members of the American Society of Breast Surgeons (ASBrS) via email [16]. Almost all respondents (97%) were familiar with the ACOSOG Z0011 study. Of those, more than half (*n* = 468, 57%) would omit ALND in patients as long as they receive whole breast irradiation, whereas 279 (36.0%) would not proceed with completion of ALND in patients who will receive accelerated partial breast irradiation after breast conservation. No difference was found in surgical attitudes among surgeons who work at academic centers and in private settings to incorporate ACOSOG Z0011 findings into their practice. In concordance with the present study and the survey in the USA, a similar survey was conducted among 799 members of the Brazilian Society of Mastology [17]. As the preferred treatment for the axilla of cN0/pSLN-positive patients compatible with the Z11 inclusion criteria, the ALND rates dropped from 99.2% before publication of the study to 47.5% in 2010 and to 18.5% in 2020 (*p* < 0.001). In patients with BCS and SLNB-micrometastasis or macrometastases, only a few surgeons (2.6% or 21.3%) would still perform ALND, whereas 30.3% or 52.2% would prefer axillary radiotherapy for the treatment of the axilla, and 67.1% or 26.5% would not suggest any additional axillary treatment, respectively. However, in those with mastectomy and nodal extracapsular extension, 43.4% and 36% of surgeons, respectively, would proceed with ALND. Similar to our findings, surgeons who did not work at academic institutes or in the capital city, who were not board-certified, and those aged ≥ 50 years were more likely to recommend ALND. In the present study, breast surgeons and surgeons with academic positions at university hospitals were more likely to be familiar with these studies and to omit ALND in patients with early breast cancer with cN0 1–2 pathologically metastatic SLNB who will receive radiotherapy.

In concordance with these studies, a survey among attendees at the annual meeting of the Association of Breast Surgeons of India (ABSI) in 2018 similarly demonstrated that only 15.8% of the surgeons had omitted completion ALND in ACOSOG Z0011 trial-eligible SLN-positive patients, whereas 45.9% would not perform a completion ALND in SLNB-positive patients with micrometastasis only [18]. Notably, 36.6% performed more than 150 breast cancer surgeries per year, and half of the respondents were surgical oncologists. The authors concluded that the low rates of axillary surgery deescalation in early breast cancer could be attributed to the unavailability of resources and lack of Indian data.

Finally, 658 patients with clinical T1-2 N0 and breast-conserving surgery (BCS) were analyzed in two groups: pre-Z0011 (*n* = 335) and post-Z0011 (*n* = 323) cohorts in a study from MD Anderson Cancer Center to assess the surgical attitudes changed after the publication of the ACOSOG Z0011 study [19]. Before ACOSOG Z0011, 85% (53/62) of SLN-positive patients underwent ALND versus 24% (10/42) after Z0011 (*p* < 0.001). Of note, intraoperative nodal evaluation decreased from 69 to 26% in the post-Z0011 period (*p* < 0.001). However, our findings have shown that the majority of surgeons in Türkiye still use intraoperative pathological assessment, unlike surgeons in the USA. This might be due to the different financial regulations in different countries.

In the recently published SENOMAC study, ALND and SLNB were compared in patients with T1-T3 breast tumors who were clinically node-negative and had one or two sentinel macrometastases. This study demonstrated that SLNB is non-inferior to ALND. Unlike the ACOSOG Z0011 and AMAROS trials, in which most patients had micrometastases, this study exclusively included patients with macrometastases and excluded those with only micrometastases. Additionally, unlike previous studies, this study included patients with T3 tumors and nodal extracapsular extension [20]. In our study, we found that approximately half of the participants indicated that they would not perform ALND in patients who had received radiotherapy after removing 2–3 sentinel lymph nodes with micro- or macrometastasis.

The second part of our survey assessed the familiarity of clinical trials regarding the feasibility and accuracy of SLNB in initially clinically node-positive breast cancer after NAC, and the impact of these trials on surgical practice. Currently, there are discrepancies in the management of the axilla in patients with cN+ breast cancer after NAC, and there is no consensus with clinically strong evidence on this subject worldwide due to the lack of published randomized trials and long-term follow-up in the last decade. Briefly, 62.4% of the respondents were familiar with ACOSOG Z1071 (*n* = 63), whereas 66.3% were familiar with SENTINA (*n* = 67). Similar to our study, 642 members of the American Society of Breast Surgeons (ASBrS) completed an anonymous online survey indicating their knowledge of ACOSOG Z1071 (86%) and SENTINA (57%). The SLNB rate increased from 45% (285/636) prior to the publication of these studies to 85% (543/638) after publication [21].

In concordance with the current report and the study of ASBrS, similar surveys have been published reflecting different surgical practices from other countries worldwide, including Brazil, Holland, and European organisations [22–25]. In the survey that was performed to assess the current axillary management after NAC among 426 Brazilian breast cancer surgeons of the Brazilian Society of Mastology, axillary complete pathologic response was necessary to omit ALND for 42.8% of responders [23]. The majority of responders (67%) indicated performing routine axillary staging by physical examination, ultrasound, and fine-needle biopsy in case of a suspicious node before NAC. Similarly, fine-needle aspiration biopsy (FNAB) or core needle biopsy of suspicious lymph nodes in USG was suggested in a patient with clinically node-positive breast cancer by 71.5% of the surgeons in the present study.

In the Dutch nationwide survey regarding the treatment of the initially cN+ axilla in patients receiving NAC among 148 surgical oncologists from November 2014 to June 2015, the majority of respondents (70%) stated that ALND could be omitted in cN+ patients with a favorable response to NAC [22]. Of these, excision of localized lymph nodes (56%) or sentinel node biopsy (SNB; 45%) could be performed in patients with a good clinical response to NAC. In the current survey, metastatic suspicious lymph nodes were marked with clips by 63.2% of the surgeons, whereas 56.4% performed TAD by wire or ROLL to retrieve the marked node after NAC.

An international European survey included 310 breast cancer specialists who were members of the European Society of Surgical Oncology and two UK-based Associations, including the Association of Breast Surgery and the British Association of Surgical Oncology. After NAC, 153/267 (57.3%) respondents routinely performed ALND, whereas 114 (42.7%) respondents performed more conservative axillary surgery with possible omission of ALND to stage the axilla. In the group with a conservative approach in those patients with a complete radiological response, the majority would perform SLNB (63%), sentinel lymph node biopsy (SLNB), excision of a previously marked positive node with SLNB (21%), and without SLNB (11%) [24].

Finally, the European Breast Cancer Research Association of Surgical Trialists (EUBREAST) reported the results of a survey regarding axillary management of cN1 patients who became ycN0 after NAC, which was completed by 349 respondents consisting of breast surgeons and radiation oncologists from 45 countries [25]. The most common axillary surgical approaches were TAD (54.2%), SLNB alone (20.9%), and ALND (22.4%). Few of them preferred targeted lymph node biopsy alone (2.5%). The majority used dual tracer as the most common technique for lymphatic mapping (62.3%). However, in the present study, use of blue dye (55.2%) was the most common mapping method followed by the dual tracer technique (42.5%), likely due to the limited availability of lymphoscintigraphy. Moreover, 70.1% of surgeons in the present report extracted at least three lymph nodes as suggested in ACOSOG Z1071, and almost all the respondents also removed clinically suspicious lymph nodes in addition to those retrieved with SLNB (94.3%, *n* = 82). In concordance with these studies, our survey demonstrated that 86% of surgeons initially performed SLNB for cN+ patients who achieved a complete clinical response after NAC, which is a higher rate than all the surveys mentioned above. Overall, more than half of the surgeons (51.5%) did not perform ALND when at least three sentinel lymph nodes were removed, and isolated tumor cells or micrometastases were detected in paraffin blocks as long as RNI was provided. However, more than half (54.5%) of the surgeons in the current study performed ALND in patients with macrometastases despite RNI, which is a lower rate than that reported in other surveys. The current standard approach is ALND in cases of residual lymph node metastasis in cN+ patients who received NAC due to the lack of randomized trials and long-term outcome data. However, our previous NeosentiTurk-MF1802 and the recently published NeosentiTurk-MF1803 study and the combined analysis of the NeosentiTurk-MF1802/MF1803 studies have shown that ALND could be avoided in meticulously selected cN+ patients who underwent SLNB after NAC with breast and/or nodal pCR, cT1-2, or low-volume residual nodal disease with luminal pathology, as long as axillary radiotherapy is provided [14, 26–28]. Therefore, the trend towards omitting ALND in cN+ after NAC among Turkish surgeons might be due to our previous studies that were presented in local and national meetings before the publication of this report.

In recent decades, less invasive surgical options have been evaluated for patients with clinically lymph node-positive breast cancer who achieve a cN0 status after NAC. With the widespread use of targeted therapies, the axillary pCR rate has increased up to 74%, making ALND unnecessary in some cases [29]. Targeted axillary dissection (TAD), which involves marking positive lymph nodes and performing SLNB with the marked lymph nodes during surgery, has emerged as an alternative to ALND. The aim of TAD is to enhance the accuracy of axillary staging and reduce the false-negative rates (FNR) associated with SLNB. In a prospective registry study conducted by Wu et al. evaluating the feasibility and oncological outcomes of TAD, no regional recurrence was observed in 85 patients in the TAD group, and 2 cases of distant recurrence were noted [30]. In our study, surgeons reported that 24.1% always and 39.1% sometimes clipped the metastatic lymph nodes before NAC. Additionally, 56.4% of patients underwent TAD by removing the marked lymph nodes using techniques such as wire localization (WL) or ROLL. Although the oncological reliability of TAD has been proven in previous studies, it is still not widely preferred by surgeons in our country. This could be attributed to the limited accessibility of these techniques.

## Conclusion

After benchmark multicentric randomized clinical trials reported no differences in local recurrence or overall survival, the trend of deescalating strategies in axillary surgery has been increasing in both initially clinically node-negative and-positive breast cancer as long as nodal radiation is provided. Educational meetings, webinars, and long-term follow-up may improve the incorporation of important prospective trials into surgical practice worldwide. The results of ongoing national and international trials are pending to reach a consensus on this subject [31–34].

## Data Availability

No datasets were generated or analysed during the current study.
